# Comparing hospital-resource utilization by an enhanced pneumonia surveillance programme for COVID-19 with pre-pandemic pneumonia admissions – a Singaporean hospital’s experience

**DOI:** 10.1099/jmm.0.001452

**Published:** 2021-12-13

**Authors:** Wenhui Huang, Gin Tsen Chai, Bernard Yu-Hor Thong, Mark Chan, Brenda Ang, Angela Chow

**Affiliations:** ^1^​ Tan Tock Seng Hospital, 11 Jalan Tan Tock Seng, 308433, Singapore; ^2^​ Lee Kong Chian School of Medicine, 11 Mandalay Road, 308232, Singapore

**Keywords:** pneumonia surveillance, COVID-19, hospital-resource utilization

## Abstract

**Introduction:**

During the early days of coronavirus disease 2019 (COVID-19) in Singapore, Tan Tock Seng Hospital implemented an enhanced pneumonia surveillance (EPS) programme enrolling all patients who were admitted from the Emergency Department (ED) with a diagnosis of pneumonia but not meeting the prevalent COVID-19 suspect case definition.

**Hypothesis/Gap statement:**

There is a paucity of data supporting the implementation of such a programme.

**Aims:**

To compare and contrast our hospital-resource utilization of an EPS programme for COVID-19 infection detection with a suitable comparison group.

**Methodology:**

We enrolled all patients admitted under the EPS programme from TTSH’s ED from 7 February 2020 (date of EPS implementation) to 20 March 2020 (date of study ethics application) inclusive. We designated a comparison cohort over a similar duration the preceding year. Relevant demographic and clinical data were extracted from the electronic medical records.

**Results:**

There was a 3.2 times higher incidence of patients with an admitting diagnosis of pneumonia from the ED in the EPS cohort compared to the comparison cohort (*P*<0.001). However, there was no significant difference in the median length of stay of 7 days (*P*=0.160). Within the EPS cohort, stroke and fluid overload occur more frequently as alternative primary diagnoses.

**Conclusions:**

Our study successfully evaluated our hospital-resource utilization demanded by our EPS programme in relation to an appropriate comparison group. This helps to inform strategic use of hospital resources to meet the needs of both COVID-19 related services and essential ‘peace-time’ healthcare services concurrently.

## Introduction

A cluster of viral pneumonia of unknown cause in Wuhan, People’s Republic of China first surfaced in December 2019. The Chinese authorities subsequently determined that the outbreak was caused by a novel coronavirus, which would later be known as severe acute respiratory syndrome coronavirus 2 (SARS-CoV-2). In Singapore, the first imported case of coronavirus disease 2019 (COVID-19) was confirmed on 23 January 2020.

Our Ministry of Health swiftly created an unequivocal, national COVID-19 suspect case definition based on the presence of severe respiratory illness and travel history to countries with high COVID-19 prevalence, or close contact with confirmed COVID-19 patients. This case definition was frequently updated to reflect both global and local epidemiological linkages.

In tandem with this, Tan Tock Seng Hospital (TTSH) – the second largest restructured hospital in Singapore employing more than 9000 healthcare workers and housing more than 1600 acute care beds – implemented the enhanced pneumonia surveillance (EPS) programme from 7 February 2020. All patients who were admitted from the Emergency Department (ED) with a diagnosis of pneumonia but not meeting the prevalent COVID-19 suspect case definition were enrolled into this programme. These patients would be tested for SARS-CoV-2 via PCR and isolated in single rooms until their results returned negative. Apart from providing an estimate of the prevalence of COVID-19 within this group of patients, this programme also mitigates inadvertent nosocomial transmission within the hospital.

The concept of a pneumonia surveillance programme is not new [1]. A broader surveillance programme based on the mere presence of respiratory symptoms but not necessarily pneumonia has also been reported [2–4]. However, there is a paucity of studies to evaluate the hospital-resource utilization of a pneumonia surveillance programme in the context of a novel viral pneumonia outbreak. With this in mind, we seek to evaluate our hospital-resource utilization demanded of the COVID-19 EPS programme.

## AIMS

Our primary aim is to compare and contrast our hospital-resource utilization of an enhanced pneumonia surveillance programme for infection detection in the early stages of a novel viral pandemic with pneumonia admissions during the corresponding pre-pandemic period.

The outcomes of interest include:

1. Incidence of ‘pneumonia’ admissions from the ED.
2. Proportion of admitted patients who subsequently tested positive for SARS-CoV-2.
3. Median length of stay (LOS).
4. Eventual discharge diagnosis.

## Methods

### Overall study design

We compared two cohorts of patients: (1) patients admitted under the EPS program in 2020 and (2) patients admitted for pneumonia during the same period in 2019.

Using de-identified data, we enrolled all patients admitted under the EPS programme from TTSH’s ED from 7 February 2020 (date of EPS implementation) to 20 March 2020 (date of study ethics application) inclusive, during the first wave of community transmission of COVID-19 infections in Singapore. April 2020 onwards brought along a second wave of COVID-19 infections, which occurred primarily in our migrant worker population who lived in dormitories and therefore had distinct demographic characteristics and risk factors. As such, the study had not included the period after March 2020.

To address the clinical question on hospital-resource utilization, we specifically looked at pertinent macroscopic data such as (a) total number of patients admitted under the EPS programme, (b) their median LOS and (c) investigations ordered. Relevant demographic and clinical data were then extracted from the electronic medical records. The primary diagnoses were obtained from the eventual electronic discharge summary.

The comparison group comprised of patients who were admitted from TTSH ED with a primary or secondary diagnosis of pneumonia, from 7 February 2019 to 20 March 2019 inclusive. A similar time period in a separate year would be crucial to control for seasonal variation of influenza.

Prior ethics approval was sought from the Domain Specific Review Board (DSRB) (DSRB reference number 2020/00319).

### Statistical analysis

Median values and their associated interquartile range were reported for non-normally distributed variables. Wilcoxon rank-sum test was then used to compare medians between the two groups. Chi square test or Fisher’s exact test was used to compare proportions between groups. Quade’s ANCOVA (non-parametric ANCOVA) was used to correct for potential confounders for non-normally distributed data. All statistical analyses were performed using SPSS Statistics version 20.0 (IBM, Armonk, NY, USA).

### EPS admission criteria – recruitment from the ED

All patients with acute respiratory symptoms presenting at TTSH’s ED were first assessed by emergency physicians. Based on their clinical assessment, coupled with other basic investigations such as a full blood count (FBC) and a chest x-ray (CXR), patients were dichotomized into two clinical entities: pneumonia versus non-pneumonia illness. The definition of pneumonia necessitated radiological consolidation in the presence of suggestive respiratory or systemic symptoms. However, emergency physicians had the liberty to exercise clinical discretion and incorporate other clinical features such as lung crepitations to make a clinical diagnosis of pneumonia, typically when radiological consolidation was equivocal.

CXRs were interpreted in real-time by emergency physicians but would eventually be reported by radiologists typically within an hour from the end of image acquisition.

Suspected COVID-19 cases were admitted directly for negative pressure, air-borne isolation, regardless of the presence of pneumonia. Patients with a primary or secondary diagnosis of pneumonia as assessed by emergency physicians, but who do not meet the prevalent COVID-19 suspect case definition, were automatically admitted under the EPS programme.

### EPS wards' configuration

Designated EPS wards have single isolation rooms. There were seven such designated EPS wards within the TTSH hospital campus – six general wards and one intensive care unit/high dependency hybrid unit which could accommodate up to 106 patients and 16 patients respectively.

### Workforce rearrangement

With the onset of COVID-19, all hospital departments with front-facing, direct patient care were segregated into ‘hot’ (covering EPS inpatients) and ‘cold’ teams (other non-EPS inpatients). Patients admitted under the EPS programme were still admitted under an appropriate subspecialty medical or surgical department based on pre-existing ED workflows. Each department has a dedicated team of doctors (i.e. ‘hot’ team) who would then manage these EPS patients exclusively. In response to an anticipated increase in manpower required for inpatient care, non-urgent outpatient appointments were generally deferred.

Each EPS ward also had a dedicated team of nurses who were re-deployed from other inpatient care areas.

Non-urgent inpatient subspecialty consults, imaging and allied health services such as physiotherapy were generally deferred until EPS patients could be safely de-isolated.

### Infection control measures

All healthcare workers used full personal protective equipment (PPE), which comprised of N95 respirators (or equivalent), face shields (or goggles), gown and gloves. N95 respirators and face shields could be reused multiple times within the same day, while gowns and gloves were discarded after single use. Full PPE was used whenever any healthcare worker has to enter a patient’s room for any reason regardless of duration. In common areas outside of the single rooms, a surgical face mask has to be donned at all times minimally. Alcohol hand rubs were also conveniently located in each isolation room.

### SARS-CoV-2 testing protocol

SARS-CoV-2 testing was typically performed via a nasopharyngeal swab by trained nurses under a standardized hospital protocol, with a second specimen collected 24 h later [5]. However, collection of lower respiratory tract specimens for SARS-CoV-2 testing, e.g. sputum was encouraged whenever feasible. All such specimens were processed in the hospital’s Department of Laboratory Medicine via quantitative real-time reverse transcription PCR (rRT-PCR) testing.

### De-isolation criteria

Patients were de-isolated when they were clinically well with at least two negative SARS-CoV-2 tests [6]. Depending on their clinical progress, they could either be discharged directly from the EPS isolation wards or they could be transferred to open cubicle wards if inpatient care was still required.

## Results

### Incidence of ‘pneumonia’ admissions and LOS)

The incidence of ‘pneumonia’ admissions from the ED was 3.2 times higher in the EPS cohort compared to the comparison cohort (*P*<0.001). However, there was no significant difference in the median LOS of 7 days (*P*=0.160) between the two cohorts [Table 1], after adjusting for age and gender using Quade’s (1967; non-parametric) ANCOVA (*F*[1,1696]=0.64; *P*=0.42) (Supplementary Material – Appendix for Quades – available in the online version of this article).

**Table 1.** Relevant clinical variables for comparison cohort (2019) and EPS cohort (2020)

	Comparison (2019) *N*=403	EPS (2020) *N*=1295	*P* value
**Overview**			
Total number of admissions	6985	6949	
Admissions with pneumonia as admitting diagnosis	403	1295	
Incidence of pneumonia (per 10 000 admissions)	577	1864	<0.001
**Demographic details**			
1. Age (years)	74 (59–83)	77 (67–85)	0.008
2. Male	190 (47.1 %)	708 (54.7 %)	0.008
**Relevant clinical outcomes**			
3. LOS (days)	7 (4–13)	7 (4–11)	0.160
4. Death at the end of hospitalization	39 (9.7 %)	93 (7.2 %)	0.102
**Radiological investigations**			
5. CXR	*N*=392 (97.3 %)	*N*=1253 (96.8 %)	
Reported as normal	9 (2.2 %)	102 (8.1 %)	<0.001
6. Chest CT imaging	*N*=44 (10.9 %)	*N*=106 (8.2 %)	0.089
**Microbiological investigations (for respiratory pathogens)**			
7. Influenza A and B PCR	*N*=299 (74.2 %)	*N*=881 (68.0 %)	
Positive (either)	27 (9.0 %)	7 (0.8 %)	<0.001
8. Respiratory virus multiplex PCR	*N*=17 (4.2 %)	*N*=56 (4.3 %)	
Positive (any organism)	4 (23.5 %)	10 (17.9 %)	0.603
9. Urinary Legionella antigen	*N*=201(49.9 %)	*N*=514 (39.7 %)	
Positive	0	0	na
10. Urinary Streptoccocal antigen	*N*=204 (50.6 %)	*N*=515 (39.8 %)	
Positive	5 (2.5 %)	12 (2.3 %)	0.923
11. Sputum AFB smear and culture	*N*=140 (34.7 %)	*N*=223 (17.2 %)	
Smear positive	10 (7.1 %)	6 (2.7 %)	0.045
Culture positive for *Mycobacterium tuberculosis *	3 (5.7 %)	0	na
12. Sputum *Pneumocystis carinii* smear	*N*=5 (1.2 %)	*N*=4 (0.3 %)	
Smear positive	0	0	na
Total performed (items 7–12)	866	2193	
**Blood investigations**			
12. WBC (x 10^9^ l^−1^)	*N*=401 (99.5 %)	*N*=1290 (99.6 %)	
Median	9.2 (7.0–12.0)	8.6 (6.5–11.9)	0.041
Range			
<4	8 (2.0 %)	55 (4.3 %)	0.036
4–10	227 (56.6 %)	746 (57.6 %)	
>10	166 (41.4 %)	489 (37.9 %)	0.210
13. C-reactive protein (mg l^−1^)	*N*=381 (94.5 %)	*N*=1188 (91.7 %)	
Median	44.8 (14.3–93.4)	24.6 (5.8–87.6)	<0.001
14. Procalcitonin (µg l^−1^)	*N*=283 (70.2 %)	*N*=854 (65.9 %)	0.111
Median	0.20 (0.08–0.49)	0.15 (0.08–0.46)	0.070

Data are presented as median (interquartile range) or numbers (%).

CT, computed tomography; CXR, chest x-ray.

### Radiology investigations – utilization and results

The overall absolute utilization of CXR and computed tomography scan (CT) of the thorax remained similar across both cohorts. However, 8.1 % of the patients in the EPS cohort had a CXR that was reported as normal, in contrast to just 2.2 % in the comparison cohort (*P*<0.001) (Table 1).

### Microbiological investigations for respiratory pathogens – utilization and results

The total number of such investigations performed was approximately 2.5 times higher in the EPS cohort compared to the comparison cohort (2193 vs. 866). The proportion of influenza PCR (0.8 % vs. 9.0 %; *P*<0.001) and sputum acid fast bacilli (AFB) smear (2.7 % vs. 7.1 %; *P*=0.045), which subsequently returned positive was significantly lower in the EPS cohort compared to the comparison cohort (Table 1).

### Haematological and biochemical investigations – utilization and results

Patients in the EPS cohort had a significantly lower median white blood cell (WBC) count (8.6 vs. 9.2×10^9^ l^−1^; *P*=0.041) and C-reactive protein (CRP) level (24.6 vs. 44.8 mg dl^−1^; *P*<0.001) compared to the comparison cohort (Table 1).

### Overall antimicrobial use

There was a significantly lower utilization rate of antibiotics typically used for community acquired pneumonia, as well as antivirals in the EPS cohort compared to the comparison cohort (Table 2).

**Table 2.** Inpatient antimicrobial use for comparison cohort (2019) and EPS cohort (2020)

	Comparison (2019) *N*=403	EPS (2020) *N*=1295	*P* value
**Antibiotics typically used for community-acquired pneumonia**			
Amoxicillin and Clavulanate	334 (82.9 %)	892 (68.9 %)	<0.001
Clarithromycin	226 (56.1 %)	405 (31.3 %)	<0.001
Azithromycin	49 (12.2 %)	43 (3.3 %)	<0.001
Ceftazidime	48 (11.9 %)	55 (4.2 %)	<0.001
Crystalline Penicillin G	46 (11.4 %)	43 (3.3 %)	<0.001
Doxycycline	45 (11.2 %)	76 (5.9 %)	<0.001
Levofloxacin	39 (9.7 %)	76 (5.9 %)	0.008
**Antibiotics typically used for hospital-acquired pneumonia**			
Piperacillin and tazobactam	126 (31.3 %)	352 (27.2 %)	0.111
Vancomycin	108 (26.8 %)	277 (21.4 %)	0.024
Meropenem	26 (6.5 %)	72 (5.6 %)	0.503
**Antiviral**			
Oseltamivir	65 (16.1 %)	107 (8.3 %)	<0.001
**Antibiotics typically used for *Pneumocystis jirovecii* pneumonia**			
Trimethoprim-sulfamethoxazole (Co-trimoxazole)	7 (1.7 %)	20 (1.5 %)	0.787
Pentamidine Isetionate (nebulized)	1 (0.2 %)	4 (0.3 %)	0.845
Clindamycin	4 (1.0 %)	23 (1.8 %)	0.272
Primaquine	1 (0.2 %)	2 (0.2 %)	0.696
**Antibiotics typically used for pulmonary tuberculosis**			
Rifampicin	8 (2.0 %)	16 (1.2 %)	0.266
Isoniazid	8 (2.0 %)	16 (1.2 %)	0.266
Ethambutol	7 (1.7 %)	16 (1.2 %)	0.477
Pyrazinamide	5 (1.2 %)	5 (0.4 %)	0.050

### Overall COVID-19 detection rate in the EPS cohort

The overall COVID-19 detection rate, defined as the presence of at least one positive SARS-CoV-2 PCR test, was 3.6 % (47 out of 1295 patients).

### Non-pneumonia discharge diagnoses

We retrieved and compared the principal diagnosis recorded in the electronic discharge summaries of both the comparison and EPS cohorts, based on SNOMED-CT codes [7]. As all patients in both cohorts were admitted from the ED with an initial diagnosis of pneumonia, we were specifically interested in patients with an eventual, principal discharge diagnosis other than pneumonia. There were a few groups of such non-pneumonia diagnoses, which occurred more frequently in the EPS cohort [Table 3]. Firstly, there was a significantly higher incidence of neurological diagnoses in the EPS cohort (1.5 vs. 0 %; *P*=0.01), in particular, cerebrovascular events. Secondly, there was also a higher incidence of fluid overload (unspecified) in the EPS cohort. Patients with fluid overload explicitly attributable to congestive cardiac failure would have been reflected accordingly as such.

**Table 3.** Selected, non-pneumonia discharge diagnoses for comparison cohort (2019) and EPS cohort (2020)

Discharge diagnosis	Comparison (2019) *N*=403	EPS (2020) *N*=1295	*P* value
**Cardiac related**	14 (3.5 %)	62 (4.8 %)	0.265
Acute myocardial infarction	4	11	
Arrhythmia	1	7	
Congestive cardiac failure	9	37	
Others*	0	7	
**Vascular related**	2 (0.5 %)	2 (0.2)	0.240
Pulmonary embolism	2	2	
**Central nervous system eelated**	0 (0 %)	19 (1.5 %)	0.01
Haemorrhagic or ischaemic stroke	0	16	
Transient ischaemic attack	0	2	
Obstructive hydrocephalus	0	1	
**Fluid overload, unspecified**	1 (0.2 %)	43 (3.3 %)	<0.001

*Others include angina, cardiac arrest, cardiomyopathy and primary pulmonary hypertension.

### Resource utilization

#### Hospital bed allocation

The total number of hospital beds set aside for our EPS programme was 122. This comprises approximately 7.3 % of our maximum bed capacity for acute care.

#### PPE use

We estimated an average of 7–8 instances per day whereby a healthcare worker has to enter a patient’s isolation room donning full PPE. The vast majority of patients were isolated for an average of 1.5 days in total. In total, anywhere between 10 000 to 15 000 sets of PPE were expanded purely to care for 1295 EPS patients.

## Discussion

Our study demonstrated that the implementation of a pneumonia surveillance programme led to a higher consumption of hospital resources. There was a 3.2 times increase in the total number of admissions for presumptive pneumonia (1295 vs. 403) but with a similar length of stay (median of 7 days). This resulted in an excess of 6244 patient-days. There was also a parallel increase in the consumption of radiological and laboratory services, leading to a commensurate increase in the strain on our healthcare system.

Pneumonia might have been over-diagnosed initially in the ED with a greater frequency in the EPS cohort compared to the comparison cohort. Firstly, there was a higher proportion of CXRs being reported as normal in the EPS cohort (8.1 % vs. 2.2 %). The unequivocal diagnosis of pneumonia requires consistent clinical and radiological findings. Therefore, a patient with respiratory symptoms but a normal CXR may not have pneumonia. Secondly, the overall lower utilization of antibiotics commonly used to treat community-acquired pneumonia, as well as antivirals in the EPS cohort suggests that hospital clinicians might have suspected upper respiratory tract infection (URTI) or even non-infectious diagnoses more frequently in the EPS cohort. Last but not least, there was a greater incidence of alternative, potentially dangerous principal diagnosis captured in the discharge summaries of our EPS cohort, which can mimick, or co-exist with pneumonia. Acute cerebrovascular events can be complicated by pneumonia [8, 9], which may then predominate the clinical consciousness in the ED. It has been estimated that approximately one in ten stroke patients experience pneumonia during the acute period of hospital care [10]. Patients with pulmonary oedema may present with similar symptoms and CXR changes as patients with pneumonia [11]. Pulmonary oedema in itself is a clinical syndrome and may have more sinister underlying aetiologies, e.g. acute myocardial infarction [12]. We have therefore identified two potentially vulnerable groups of patients with specific diagnostic and dispositional challenges in the ED; and precisely in whom close monitoring in isolation rooms might be challenging.

As of 20 March 2020, our EPS programme detected COVID-19 in 3.6 % (47/1295) of all EPS patients. At a national level, this corresponded to 12.2 % (47/385) [13] of all confirmed COVID-19 cases. All confirmed COVID-19 cases were managed in restructured hospitals until they were deemed non-infectious in order to mitigate community spread, regardless of initial illness severity. Singapore was able to do this due to the relatively low prevalence of COVID-19 and the adequacy of our collective healthcare infrastructure. At that point in time, patients were generally tested only when they had consistent symptoms, or if they were close contacts of confirmed cases.

Whether our low detection rate justified the implementation of such an extensive surveillance programme cannot be addressed by this study. This is a complex question, which takes into account population factors, viral factors and healthcare system factors. It is worth noting that identification of causative pathogen in patients with radiologically confirmed pneumonia has only been estimated to be 38 % in the pre-COVID-19 era [14].

## Potential directions to refine our EPS program

Our EPS programme was intended as a second-tier, screening tool to complement testing of suspect cases, during a time period in which the overall number of COVID-19 positive cases were low nationwide. Therefore, the pick-up rate was expected to be low in relation to the resources required.

To mitigate hospital spending in inappropriate patient groups with an alternative principal diagnosis other than pneumonia, there are a few measures that can be undertaken. Early radiologist reporting of CXRs taken in ED can be implemented and reinforced. Inpatient teams may also be empowered to de-isolate patients with a clear, alternative diagnosis earlier – perhaps after only one negative COVID-19 swab.

In healthcare systems in which there are insufficient isolation beds to accommodate all patients with pneumonia, vaccination status may also be incorporated as a downstream, risk stratification tool given its reported efficacy [15, 16].

## Study’s limitation

There are several limitations of our study. Firstly, we do not have data on the co-morbidities of all the patients in both cohorts. Secondly, we also do not have longitudinal follow-up data in terms of re-admissions, as well as 6 month and 1 year mortality. Thirdly, we are unable to accurately determine the exact financial cost sustained by the EPS programme. Lastly, we are unable to compare overall hospital-resource utilization beyond COVID-19 related admissions between both cohorts since there were changes to national ambulance conveyance, which occurred in March 2020.

## Conclusion

Our study successfully evaluated our hospital-resource utilization demanded by our EPS programme in relation to an appropriate comparison group. From the perspective of hospital administrators, the results of our study help to inform strategic use of hospital resources to meet the needs of both COVID-19 related services as well as essential ‘peace-time’ healthcare services concurrently, especially given the global backdrop of multiple COVID-19 waves. From the perspective of hospital clinicians, the results of our study highlight two potential vulnerable groups of patients in whom heightened clinical vigilance may be prudent.

## Supplementary Data

Supplementary material 1
