# Supplementary material for: Comparing hospital-resource utilization by an enhanced pneumonia surveillance programme for COVID-19 with pre-pandemic pneumonia admissions – a Singaporean hospital’s experience
Source: J Med Microbiol. 2021 Dec 13;70(12):001452. doi: 10.1099/jmm.0.001452 (PMC8744277; doi:10.1099/jmm.0.001452)
Supplement: Supplementary material 1 [file jmm-70-1452-s001.pdf]

Supplementary Material - Appendix

Variables

- LOS (continuous)
    - Visually noted right skew
    - Shapiro-Wilk tests of normality suggests non-normal distribution (*df* 1698; *stat* 0.64; $p < .001$)
- Age (continuous)
    - Visually mild left skew
    - Shapiro-Wilk tests of normality suggests non-normal distribution (*df* 1698; *stat* 0.94; $p < .001$)
- Male (898 vs. 800 female)
    - Not associated with LOS based on Mann-Whitney U Tests ($Z = -0.86$; $p = .39$)
- Intervention (1295 vs. 403 control)
    - Associated with gender based on Chi-Square Tests (χ^2 [1,1698] = 6.99, $p = .009$)
    - Associated with age based on Wilcoxon Signed Rank Tests ($Z = -35.63$; $p < .001$)

Median LOS of both Intervention and Control groups = 7 days
However, intervention is significantly associated with LOS based on Mann-Whitney U Tests ($Z = -2.07$; $p = .039$).

In view of the associations between the intervention and covariates, Quade's (1967; non-parametric) ANCOVA was performed, which revealed non-significant associations in LOS between intervention and control groups having controlled for age and gender (F[1,1696] = 0.64, $p = .42$).

References

Quade, D. (1967). Rank analysis of covariance. *Journal of the American Statistical Association*, *62*(320), 1187–1200. https://doi.org/10.1080/01621459.1967.10500925
